# The SARS-CoV-2 vaccination rate and hesitation in Shanghai older adults with dementia

**DOI:** 10.3389/fpubh.2023.1172642

**Published:** 2023-06-27

**Authors:** Yang Yang, Jing Nie, Fei Sun, Jinghua Wang, Jianhua Chen, Ling Li, Meiqing Sheng, Sijie Yang, Lei Yu, Xia Li

**Affiliations:** ^1^Alzheimer’s Disease and Related Disorders Center, Department of Geriatric Psychiatry, Shanghai Mental Health Center, Shanghai Jiao Tong University School of Medicine, Shanghai, China; ^2^School of Social Work, Michigan State University, East Lansing, MI, United States

**Keywords:** dementia, older adult, SARS-CoV-2 vaccine, hesitation, omicron

## Abstract

**Background:**

Older adults, particularly those with dementia, are at the greatest risk for being affected by SARS-CoV-2. Despite the Chinese government’s efforts to encourage older adults to receive SARS-CoV-2 vaccines, the vaccination rate, especially among older adults with dementia, remains low.

**Objective:**

This study aimed to examine the willingness and attitudes towards vaccination among guardians of older adults with dementia and to uncover the factors that may have influenced attitudes towards vaccination during the 2022 Omicron Variant of SARS-CoV-2 outbreak in Shanghai, China.

**Methods:**

We conducted a cross-sectional study using self-administered anonymous questionnaires to guardians of dementia patients in three settings: psychogeriatric inpatient wards, long-term care facilities, and home settings from April to May 2022. The primary outcome was participants’ willingness to allow dementia patients to receive SARS-CoV-2 vaccines. Logistic regression analyses were used to identify factors associated with vaccination willingness.

**Results:**

A total of 327 valid questionnaires were collected. The vaccination rate among participants from long-term care facilities (12.9%) was lower than those in the psychiatric ward (19.3%) or community-dwelling settings (27.1%) (*p* < 0.05). The guardians’ primary concern was that vaccination would aggravate the health conditions of dementia patients [adjusted odds ratio (OR) = 5.11; 95% confidence interval (CI): 1.86–14.05]. Additionally, negative reports about the vaccination [OR = 3.94; 95% CI: 1.68–9.24], and adverse reactions [OR = 2.50; 95% CI: 1.13–5.52] were related to higher odds of vaccine hesitancy.

**Conclusion:**

Our results showed that low vaccination rates in older adults with dementia were mainly due to their guardians’ concerns about vaccine safety. Our findings first uncovered the actual SARS-CoV-2 vaccination rates among older adults with dementia and may provide potential interventions to reduce unjustified worries towards vaccination.

## Introduction

Omicron, which emerged at the end of November 2021 with a rapid transmission and mutation rate, is the current variant of SARS-CoV-2 (1, 2). It has a medical, public health, and economic crisis worldwide (3, 4). Although novel antiviral agents, such as molnupiravir (5), have been developed, vaccination remains the most effective way to prevent and control the spread of Omicron (6). As the geriatric population usually has various chronic diseases, some individuals experience a very challenging clinical course after infection by SARS-CoV-2. Therefore, vaccination for the older population is prioritized in most countries (7, 8). High vaccination rates can protect both vaccinated and unvaccinated populations by creating herd immunity and reducing the chance of virus mutation. However, people who are reluctant to receive the SARS-CoV-2 vaccine pose a barrier to achieving herd immunity.

The Omicron pandemic has made profound health and socioeconomic impacts on residence in Shanghai since March 2022 (9–11). Although Omicron has a lower chance of developing serious respiratory syndrome, the case fatality ratio (CFR) is still high in the unvaccinated older population (12). In Shanghai, the most populous metropolitan area in China, the local vaccination rates of the older adult population are subpar. As of May 22nd, the fully vaccinated rate for people above 60 years old in Shanghai was 62.11%, and the booster coverage rate was 39.26% (13, 14).

Dementia patients are more vulnerable to SARS-CoV-2 due to their advanced age and high prevalence of comorbidities such as cardiovascular diseases and diabetes (15–18). Moreover, people with dementia usually lack the ability to self-isolate (19–21) or to understand and follow public health guidelines, such as maintaining good hygiene, social distancing, and wearing protective masks (22), due to their cognitive impairment and/or mental behavioral symptoms (23, 24). Previous studies have shown that people with dementia are twice as likely to be at risk for SARS-CoV-2 compared to the general older adult population (25). Therefore, vaccination is particularly important for this group of people, but studies are limited.

To date, there has been a lack of data on SARS-CoV-2 vaccination willingness and coverage among people with dementia in China. This information is urgently needed to understand the current vaccination situation and facilitate more efficient vaccination strategies. In this study, we report the vaccination coverage of people with dementia, their guardians’ willingness, and reasons for hesitancy, in order to provide evidence to inform vaccination strategies.

## Materials and methods

### Study design and sample

A cross-sectional study was conducted among guardians of older adults with dementia in Shanghai, China, between April and May 2022.

Guardians are defined as family members who are responsible for making decisions for the person living with dementia. Data was collected from guardians of older adults with dementia from three settings: psychiatric wards, long-term care facilities, and community-dwelling settings through the Questionnaire Star website^1^ and online questionnaires using a convenient sampling method. Respondents were informed that their participation was voluntary, and consent was implied upon completion of the questionnaire. Criteria for inclusion of respondents consisted of: (1) Guardians who can decide on vaccination for an older relative diagnosed with dementia and (2) Guardians who must be older than 18 years of age and able to provide informed consent. Exclusion criteria included: (1) Duplicate answers, (2) answer time less than 90 s, and (3) incomplete questionnaires (with more than 20% missing data). A total of 400 questionnaires were distributed and 327 completed surveys were received.

### Measures

The first draft of the questionnaire was formed through literature review and family interviews, and the dimensionality of the questionnaire entries was assessed by three specialists, one nurse and two social workers at the Shanghai Mental Health Center, respectively. It was also tested by two non-researchers to ensure that the entries were comprehensible. The questionnaire is in simplified Chinese so that guardians can fully understand all entries.

The first part of the questionnaire collected demographic and sociological information, include the role of guardian (couple = 1; adult children = 2; non-immediate family = 3), age (≤60 = 1; >60 = 2), gender (male = 1; female = 2), education (Junior Secondary and below = 1; High school = 2; University or above = 3), as well as the patient’s age (60–69 = 1; 70–79 = 2; 80–89 = 3; over 90 = 4), chronic diseases in older adults (no = 1; 1kind = 2; 2 or more = 3) and whether the patients been vaccinated against SARS-CoV-2.

The second part of the questionnaire collected the respondents’ willingness to let the older adults with dementia receive the SARS-CoV-2 vaccine.

Dependent Variable. If the patient is not vaccinated, the patient’s willingness to let patients receive the SARS-CoV-2 vaccine was assessed using one item: “Do you want the patient in the hospital to be vaccinated against SARS-CoV-2?” The items are divided into two categories: willing(code as 0) or hesitant(code as 1).

Independent variables. The worry about vaccination, contains eight items. “Greater susceptibility of older patients to the virus, “Greater illness severity for older patients”， “Greater illness severity for patients with other underlying conditions, “Greater illness severity for unvaccinated patients, “side effects from the vaccine, “Negative interactions between the vaccine and current prescribed medications”， “Concern about the vaccine causing dementia worse, “Concern derived from negative messaging from various sources about vaccination.” The items are coded as Yes(1) and No(0).

Attitudes towards vaccines consist of five items. “Trust of the vaccine’s safety,” “Opinion of the vaccine efficacy, “Opinion of the vaccine’s capability to prevent disease, “The reduction of possibility of severe illness, “Dementia as a contraindication for vaccination.” The items are divided into three categories: Yes (code as 3), hesitant (code as 2), and No (code as 1).

The questionnaire was distributed on social media family groups and newsletters for clients. Informed consent was embedded at the beginning before of the questionnaire. To ensure data quality and the validity of the responses, we received some responses and then adjusted the settings of the questionnaire. First, the questionnaire was only accessible to participants who provided a cell phone number, and participants were unable to complete the questionnaire more than once. Secondly, in the questionnaire, we emphasized that eligible respondents must be guardians who oversee a dementia patient, that is, making decisions for the dementia patient, including vaccination.

### Statistical analysis

Statistical analysis was performed using SPSS 24.0. Measurement data were profiled by frequency and percentages, Chi-square test (*χ*^2^) was used to compare differences between groups, and chi-square *post hoc* test was used for differential results. A logistic regression equation was constructed with Willingness to vaccinate as dependent variable: The first group was those who were willing to vaccinate, coded as 0. The second group included people who were hesitant, coded as 1. The independent variables were: guardians’ education, patient’s age and worry about vaccination and attitudes towards vaccines containing 13 items. *p* < 0.05 was considered statistically significant. GraphPad Prism was used for mapping.

### Ethical considerations

This study was carried out in accordance with the recommendations of the “Shanghai Mental Health Center ethical standards committee on human experimentation” with consent from all subjects. The protocol was approved by the “Shanghai Mental Health Center ethical standards committee.”

## Results

### Study sample characteristics

A total of 327 participants joined the Guardian Survey. Among them, 88 had a dependent from hospitals, 132 from long-term care facilities, and 107 from families. Close to three-fourths of guardians (*n* = 244, 74.6%) were mainly adult children. Most of the study participants were age ≤60 years (67.3%), female (66.7%), and had a university degree or higher (72.2%). Most adults with dementia (40.1%) were between the ages of 70 and 79. The percentage of dementia patients with one or more underlying health conditions was 39.1 and 22.6%, respectively.

There was no significant difference in guardian status, age, and age of the older adult with dementia under different sources (*p* > 0.05). However, there were statistical differences between the three groups in terms of guardian sex (*x*^2^ = 14.95, *p* = 0.001), level of education (*x*^2^ = 11.27, *p* = 0.024) and the number of underlying diseases in older adults (*x*^2^ = 18.45, *p* = 0.001).

There were a gender differences between the guardians of the older adult at home and the guardians of the older adult in long-term care facilities and hospitals. For example, the proportion of female guardians among the older adult at home was significantly larger than in long-term care facilities and hospitals. There was a significant difference between home and long-term care facilities in the educational attainment of guardians and whether older people with dementia had underlying health conditions. Guardians of older adults living at home were significantly more educated than those in long-term care facilities. The proportion of people without underlying diseases was lower than those in long-term care facilities. There was no significant difference between the remaining groups. Details of each group are provided in Table 1.

**Table 1:** Characteristics of guardians and patients in the sample.

	Total (*n* = 327)	Hospital (*n* = 88)	Long- term care (*n* = 132)	Home (*n* = 107)	χ^2^	*p*
**Guardians, n(%)**						
Couples	63 (19.3)	18 (20.5)	28 (21.2)	17 (15.9)	3.20	0.525
Adult children	244 (74.6)	64 (72.7)	94 (72.1)	86 (80.4)		
Nonimmediate family	20 (6.1)	6 (6.8)	10 (7.6)	4 (3.7)		
**Age, n(%)**						
≤60	220 (67.3)	61 (69.3)	84 (63.6)	75 (70.1)	1.35	0.51
>60	107 (32.7)	27 (30.7)	29 (36.4)	36 (29.9)		
**Gender, n(%)**						
Female	218 (66.7)	47 (53.4)	86 (65.2)	85 (79.4)^#^	14.95	***0.001***
**Education, n(%)**						
Junior Secondary and below	19 (5.8)	9 (10.2)	6 (4.5)	4 (3.7)	11.27	***0.024***
High school	72 (22.0)	18 (20.5)	38 (28.8)^*^	16 (15.0)		
University or above	236 (72.2)	61 (69.3)	88 (66.7)^*^	87 (81.3)		
**Guardian vaccinated COVID-19 vaccine, n(%)**						
Yes	273 (83.5)	74 (84.1)	106 (80.3)	93 (86.9)	1.91	0.386
No	54 (16.5)	14 (15.9)	26 (19.7)	14 (13.1)		
**Patients, n(%)**						
**Age, n(%)**						
60-69	57 (17.4)	21 (23.9)	24 (18.2)	12 (11.2)	8.42	0.209
70-79	131 (40.1)	34 (38.6)	55 (41.7)	42 (39.3)		
80-89	112 (34.3)	29 (33.0)	40 (30.3)	43 (40.2)		
Over 90	27 (8.3)	4 (4.5)	13 (9.8)	10 (9.3)		
**Chronic health diseases, n(%)**						
NO	125 (38.2)	37 (42.0)	41 (31.1)	47 (43.9)	18.45	***0.001***
1 kind	128 (39.1)	26 (29.5)	70 (53.0)^*^	32 (29.9)		
2 or more	74 (22.6)	25 (28.4)	21 (15.9)	28 (26.2)		

*significantly different compared to home; ^#^significantly different compared to hospital, long-term care.

### Vaccination rate of people with dementia by care setting

**Figure 1:**
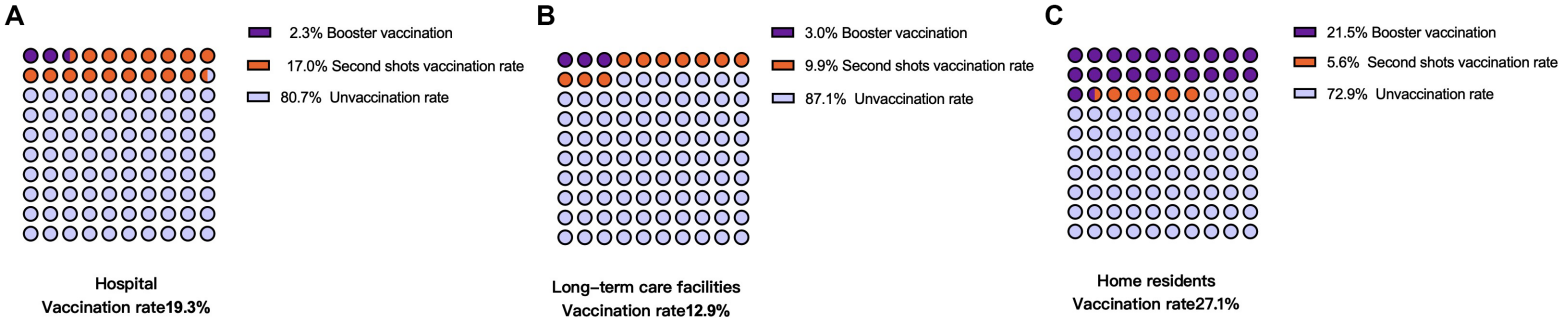
Vaccination rate of people with dementia by care setting.

In Figure 1, the overall vaccination rate was highest among the community living older adults at 27.1%, followed by hospitals at 19.3%, and long-term care facility at 12.9%. The highest percentage of booster immunizations completed was among older adults at home at 21.5%, followed by those in long-term care at 3.0%, and hospitals at 2.3%. The completion rate of two doses was 17.0% in hospitalized older adults, higher than those in the other two groups. See Figure 1 for more details.

### Guardians planning future vaccination options

A total of 260 patients were not vaccinated in the study, with 42% of guardians willing to vaccinate patients in the future and 58% expressing hesitation. There were no significant differences between the two groups related to the guardians, age, sex, and presence of underlying diseases in dementia patients (*p* > 0.05).

Moreover, there were significant differences in guardian vaccinated COVID-19 vaccine(*x*^2^ = 12.98, *p* = 0.00). The age of patients with dementia had different effects on the guardian’s willingness to vaccinate (*x*^2^ = 5.44, *p* = 0.14). Details of each group are provided in Table 2.

**Table 2:** Characteristics of the sample group without vaccination.

	Total (*n* = 260)	Willing (*n* = 109)	Hesitation (*n* = 151)	χ^2^	*p*
**Caregivers, n(%)**					
Couples	50 (19.1)	21 (19.3)	29 (19.2)	2.08	0.35
Adult children	191 (73.5)	83 (76.5)	108 (71.5)		
Nonimmediate family	19 (7.3)	5 (4.6)	14 (9.3)		
**Age, n(%)**					
≤60	177 (68.1)	74 (67.9)	103 (68.2)	0.003	0.95
>60	83 (31.9)	35 (32.1)	48 (31.8)		
**Gender, No.(%)**					
Female	168 (64.6)	69 (63.3)	99 (65.6)	0.14	0.70
**Education, n(%)**					
Junior Secondary and below	9 (3.4)	3 (2.8)	6 (4.0)	0.28	0.86
High school	62 (23.9)	26 (23.9)	36 (23.8)		
University or above	189 (72.7)	80 (73.4)	109 (72.2)		
**Guardian vaccinated COVID-19 vaccine, n(%)**					
Yes	212 (81.5)	100 (91.7%)	112 (74.2)	12.98	***0.00***
No	48 (18.5)	9 (8.3%)	39 (25.8)		
**Patients, n(%)**					
**Age, n(%)**					
60-69	44 (16.9)	20 (18.3)	24 (15.9)	5.44	0.14
70-79	97 (37.3)	47 (43.1)	50 (33.1)		
80-89	95 (36.5)	31 (28.4)	64 (42.4)		
Over 90	24 (9.2)	11 (10.1)	13 (8.6)		
**Chronic health diseases, n(%)**					
NO	97 (37.3)	43 (39.4)	54 (35.8)	0.36	0.83
1 kind	104 (40.0)	42 (38.5)	62 (41.1)		
2 or more	59 (22.7)	24 (22.0)	35 (23.2)		

### Vaccination intentions related to vaccination apprehension and vaccine knowledge

**Figure 2:**
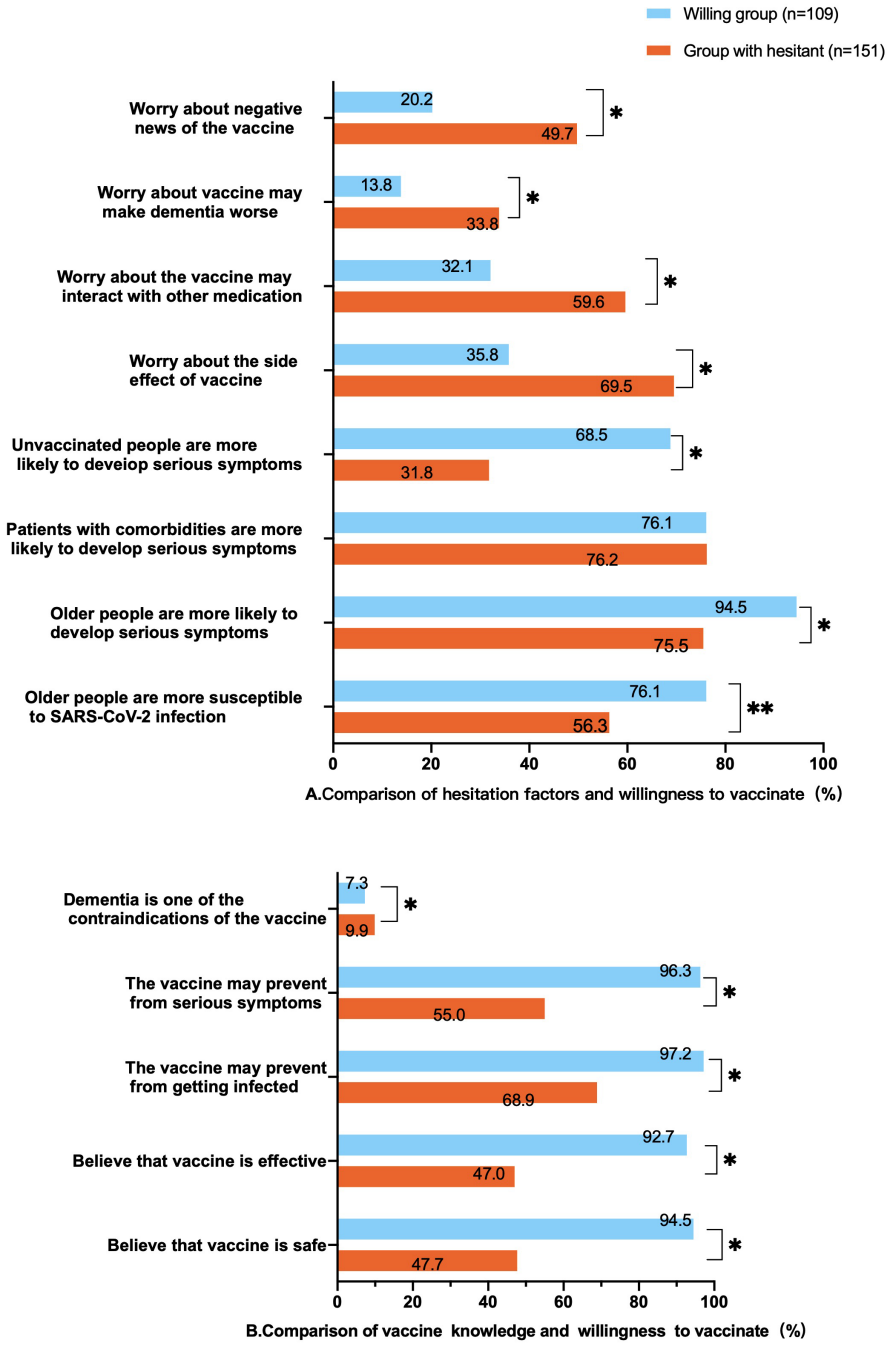
Hesitancy and vaccine knowledge compared to willingness to get vaccinated.

Significant differences were found between the consent and hesitation groups for the options of “guardian concerns about negative vaccine news,” “vaccination aggravates cognitive impairment,” “vaccine-induced drug interactions,” and “concerns about vaccine side effects.” Among them, the percentage of hesitation group was significantly higher than the consent group. Differences were shown between the options “unvaccinated people are more likely to develop serious symptoms,” “older people are more likely to develop serious symptoms,” and “older people are more susceptible to SARS-CoV-2 infection.” See Figure 2 for more details.

### Factors influencing guardians’ vaccination intentions for older adults with dementia

**Figure 3:**
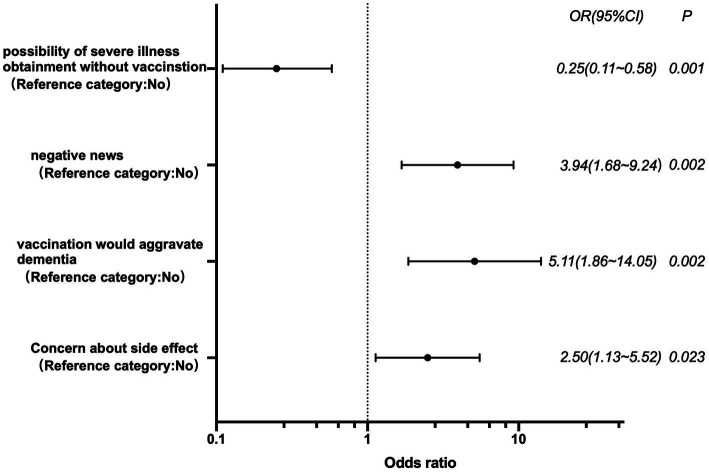
Logistic regression analysis of influencing factors of vaccination hesitancy.

Logistic regression was used to investigate the factors affecting guardians’ hesitation. The results showed that the main factors of vaccination hesitation were guardians’ concern about side effects of the vaccine (OR = 2.50, 95% CI:1.13–5.52, *p* = 0.023), worsen dementia by the vaccine (OR = 5.11, 95% CI:1.86–14.05, *p* = 0.002), negative news about the vaccine(OR = 3.94, 95% CI:1.68–9.24, *p* = 0.002). However, those who thought possibility of severe illness obtaining without vaccination was higher were more willing to agree to the person with dementia being vaccinated (OR = 0.25, 95% CI: 0.11–0.58, *p* = 0.001) Figure 3.

## Discussion

Our study revealed that 27.1% of people with dementia living at home were vaccinated, with the lowest vaccination rate of 12.9% found among those residing in long-term care settings. The vaccination completion rate for older adults was influenced by guardian education; higher vaccination rates were observed among guardians with junior middle school education or below, while lower rates were seen among those with a college degree or above. Patients aged between 70 and 79 without chronic diseases were more likely to have their families complete the vaccination process. The willingness of guardians to vaccinate patients significantly improved with medical support, indicating that even when a patient is acutely hospitalized for dementia, it does not impact the family’s decision to vaccinate the older adult (See Figure 3).

Guardians who did not arrange for vaccinations for the older adults cited concerns such as negative news about the vaccine, vaccine-induced drug interactions, vaccine side effects, the exacerbation of dementia due to vaccination, and the belief that dementia was a contraindication for vaccination. Guardians were less likely to choose vaccination for patients aged 80 and above in the future. The willingness to not give vaccines to patients with dementia in the future was 81.3% for those whose guardians had not vaccinated COVID-19 vaccine. For those whose guardians were vaccinated themselves, the willingness to not give vaccines to patients with dementia in the future was 52.8%.

Our findings, which showed that only 19.2% of older adults with dementia were vaccinated, revealed disparities when compared to national data in China or citywide date in Shanghai. Nationwide, 86.44% of individuals over 60 are fully vaccinated in China (26) and the number is 62.11% in Shanghai. This proportion of vaccinated dementia in Shanghai is much lower compared to the 91.2% of individuals in the United States (27). This may be attributed to the specific characteristics of people with dementia, whether living at home or in long-term care facilities, who require a guardian to make appointments by proxy, sign consent forms for vaccination, and accompany them to designated vaccination locations.

Vaccines play an important role in reducing the incidence of SARS-CoV-2 and related deaths among older adults with dementia (28). However, this study indicates that Shanghai has the lowest vaccination rate among dementia patients in long-term care facilities, at 12.9%. In comparison, 81.4% of nursing home residents in the United States and 78.2% in some nursing homes in France have been vaccinated (29, 30), much higher than the rates for dementia patients in our long-term care facilities. This disparity may be due to the stigma attached with dementia as a disease (31), challenges in caregiving, and difficulties coordinating the vaccination process. Our results further demonstrated that guardians were more likely to express passive willingness to vaccinate with medical support rather than active willingness to vaccinate.

With dementia patients unable to make vaccination decisions for themselves due to the nature of their disease, the guardian’s will becomes increasingly important. The study results demonstrated that among all age groups, families were most willing to vaccinate patients aged 70 to 79, while relatives were least willing to vaccinate patients aged 80 to 89. Additionally, guardians younger than 60 were more likely to choose vaccination than those older than 60. Our team has previously shown that older adults’ concerns about outbreaks fluctuate over time. They were not very worried at the beginning of the pandemic, but their concerns have gradually increased as the pandemic has continued (32). This may explain why older guardians in our study were less willing to vaccinate people with dementia. Furthermore, older adults have greater confidence in the government’s pandemic control strategy (33). The reasons for hesitating to vaccinate were similar to those of other young adults and older people in Shanghai (34, 35), such as exposure to negative news about the vaccine, concerns about vaccine-induced drug interactions, and worries about side effects.

The European Alzheimer’s Association has been advocating for prioritizing dementia patients to receive the SARS-CoV-2 vaccine (36). However, our study reveals that the perception of vaccinations exacerbating dementia or that dementia is a contraindication to vaccinations remains a major concern for families of people with dementia. This suggests that we need to target vaccinations for people with dementia at a later stage, involving medical professionals to communicate the benefits and provide support for the vaccination process.

Fifty-eight percent of dementia patients were not vaccinated, and their guardians did not intend to vaccinate them in the future. In a separate study of SARS-CoV-2 vaccinations among Shanghai residents, participants were more reluctant to vaccinate older adults in their homes, similar to our findings (37). Guardians of people with dementia are hesitant to vaccinate due to concerns about vaccine side effects, negative news, and the perception that vaccinations exacerbate dementia. Emphasizing the necessity and safety of SARS-CoV-2 vaccinations among guardians and the need for the public health sector to better understand and interpret vaccination contraindications for such older individuals is crucial.

To our knowledge, the current study was the first to report vaccination rates in older adults with dementia and the influential factors for guardians’ vaccination decisions. Our results are noteworthy for understanding the reasons behind vaccination hesitancy among guardians of older adults with dementia, enabling the promotion and popularization of scientific knowledge to increase guardians’ willingness to vaccinate those with dementia.

Like all studies, ours had several limitations. Firstly, we did not investigate the potential impact of vaccine types on the willingness to get vaccinated. Data on the efficacy of various vaccines for this specific population was not available at the time of the questionnaire distribution. In future surveys, we will include the vaccine type preference as a factor. Secondly, our data sample was drawn from the geriatric department of the Shanghai Mental Health Center, the cognitive impairment care center, and guardians of homebound patients. All these patients had received a confirmed diagnosis of dementia after proactively seeking medical care. Given the relatively low rate of dementia consultations in China, this cohort may not be representative of all dementia patients. These families, who have sought medical assistance, likely possess a higher understanding of dementia and express a greater concern for the adults. Additionally, we conducted this survey in Shanghai, a region with generally higher educational levels than other parts of China. Therefore, our data might inherently be skewed and may not truly represent the views of all guardians of dementia patients. Thirdly, we conducted the survey through an online questionnaire, which was voluntary, creating potential for bias in the responses. Guardians’ preferences may change over time, and this might not be reflected in the original survey responses. In future studies, we plan to directly engage the guardians of dementia patients, addressing their real concerns regarding vaccination through face-to-face, semi-structured interviews.

## Conclusion

Vaccination rates for SARS-CoV-2 are notably low among people with dementia, particularly those residing in long-term care facilities. With the support of medical supervision, hesitancy among guardians can be significantly reduced, resulting in 42% of guardians potentially opting for vaccination later. Factors such as the patient’s age, vaccine concerns, and misperceptions contribute to vaccine hesitancy among family guardians. Our findings shed light on the low vaccination rates observed among older individuals with dementia in Shanghai and can help inform future policy and practice initiatives aimed at promoting vaccination in this vulnerable population.

## Data availability statement

The original contributions presented in the study are included in the article/supplementary material, further inquiries can be directed to the corresponding authors.

## Ethics statement

The studies involving human participants were reviewed and approved by This study was carried out in accordance with the recommendations of the “Shanghai Mental Health Center ethical standards committee on human experimentation” with consent from all subjects. The protocol was approved by the “Shanghai Mental Health Center ethical standards committee.” The patients/participants provided their written informed consent to participate in this study.

## Author contributions

XL, LY, and YY conceived and designed the study. YY collected and analyzed data. YY and JN drafted the manuscript. FS, JW, JC, LL, MS, and SY collected data and provided feedback on the manuscript. All authors contributed to the article and approved the submitted version.

## Funding

This study was supported by Shanghai Fifth Round of Public Health Three-year Action Plan (GMV - 9.2), Clinical Research Center Project of Shanghai Mental Health Center (CRC2021ZC01), and study project of Shanghai Mental Health Center (2022-YJ07).

## Conflict of interest

The authors declare that the research was conducted in the absence of any commercial or financial relationships that could be construed as a potential conflict of interest.

## Publisher’s note

All claims expressed in this article are solely those of the authors and do not necessarily represent those of their affiliated organizations, or those of the publisher, the editors and the reviewers. Any product that may be evaluated in this article, or claim that may be made by its manufacturer, is not guaranteed or endorsed by the publisher.

## Footnotes

^1^ https://www.wjx.cn
